# Plant Breeding Notes: A New Article Type for *Plant‐Environment Interactions*

**DOI:** 10.1002/pei3.70067

**Published:** 2025-06-17

**Authors:** Jonathan Ingram, Gareth B. Jenkins, Abdelbagi Ismail, Jens Léon, Frank Ordon

**Affiliations:** ^1^ John Wiley & Sons Inc UK; ^2^ International Rice Research Institute Kenya Nairobi Kenya; ^3^ Rheinische Friedrich‐Wilhelms‐Universitat Bonn Institut fur Nutzpflanzenwissenschaften und Ressourcenschutz Bonn Germany; ^4^ Julius Kuhn‐Institut Bundesforschungsinstitut fur Kulturpflanzen Quedlinburg Germany

1

Plant breeding covers a wide range of research critically important for the advancement of agriculture worldwide. In‐depth research articles usually form the version of record for disseminating new developments, but for more limited, sometimes highly specialist studies, a simpler research note may be sufficient and more appropriate. This is the thinking behind a new short article category for *Plant‐Environment Interactions*, Plant Breeding Notes, with the aim of bringing useful information for plant breeders to publication simply and quickly for our authors.

The general approach follows similar situations in other titles in adjacent fields, including *Ecology and Evolution* where Nature Notes (Moore et al. 2020) and Genetics Notes (Jenkins et al. 2024) have proved popular with authors; *Aquaculture*, *Fish and Fisheries* with Underwater Notes (Bailey et al. 2024); *Cell Biochemistry and Function* with Cell Bio Notes (Gaudin and Heath 2025); and most recently *Environmental Microbiology Reports* with Microbiome Notes (Jenkins et al. 2025).

Situations where Plant Breeding Notes might be appropriate include:

1. Trials covering smaller numbers of sites or carried out for a limited period, providing a valuable preliminary information resource leading into more comprehensive, usually multi‐environment (or multi‐year) projects;
2. Highly specialized, regional studies, important locally but with limited application elsewhere;
3. Work which, although confirmatory, is still useful in validation of independent studies;
4. And descriptive genotype analyses (i.e., without further evaluation in plant improvement, but providing a starting point for such work).

This new short article category has been partly developed based on the increasing numbers of manuscripts which we have received containing potentially useful data, but not fulfilling the requirements of full research articles in this field where more detailed analysis is needed. Assessment will of course follow our usual practice, including full peer review, ensuring quality and a focus on constructive approaches to publication—we look for reasons to accept, not to reject.

Concise presentation is encouraged, which includes critical information only, that is, a brief introduction, key methodology which allows replication by a third party, and results, with the latter usually combined with any essential discussion points such as consideration beyond the local context, limitations, and next steps. Supporting Information can be used for additional data.

Reliable data is vital, and any genetic material must be accessible. The International Nucleotide Sequence Database Collaboration (INSDC: DDBJ/GenBank/ENA‐EBI) is our recommended repository for sequence data, but any repository chosen must be freely available without the need to request access and use a permanent identifier (i.e., a DOI). Further information is provided in our Author Guidelines, and we suggest minimum standards (Jenkins et al. 2023) are followed to make sure any data provided and archived follows FAIR principles (Findable, Accessible, Interoperable, Reusable; Wilkinson et al. 2016).

We also continue to welcome referrals of longer research articles from other titles. In this, the journal *Plant Breeding* is an important partner, covering all areas of plant improvement, including breeding methodologies and genetics. Where articles do not reach the high novelty criteria for *Plant Breeding* but still provide valuable research for the community, they will be offered the option of publication in *Plant‐Environment Interactions*, and we will be pleased to take these forward as long as the methodology is reliable. Peer review is guaranteed for referrals from *Plant Breeding*. As per our journal philosophy, the work we publish builds on our understanding, but immediate impact is not a criterion for acceptance. We always look to work with authors to help get their work out there, and this will remain the case for these papers.

## Conflicts of Interest

The authors declare no conflicts of interest.

## Data Availability

The authors have nothing to report.
